# APOBEC3 versus Retroviruses, Immunity versus Invasion: Clash of the Titans

**DOI:** 10.1155/2012/974924

**Published:** 2012-06-06

**Authors:** Ann M. Sheehy, Julie Erthal

**Affiliations:** ^1^Department of Biology, College of the Holy Cross, Worcester, MA 01610, USA; ^2^Department of Biology, Clark University, 950 Main Street Worcester, MA 01610, USA

## Abstract

Since the identification of APOBEC3G (A3G) as a potent restriction factor of HIV-1, a tremendous amount of effort has led to a broadened understanding of both A3G and the APOBEC3 (A3) family to which it belongs. In spite of the fine-tuned viral counterattack to A3 activity, in the form of the HIV-1 Vif protein, enthusiasm for leveraging the Vif : A3G axis as a point of clinical intervention remains high. In an impressive explosion of information over the last decade, additional A3 family members have been identified as antiviral proteins, mechanistic details of the restrictive capacity of these proteins have been elucidated, structure-function studies have revealed important molecular details of the Vif : A3G interaction, and clinical cohorts have been scrutinized for correlations between *A3* expression and function and viral pathogenesis. In the last year, novel and unexpected findings regarding the role of A3G in immunity have refocused efforts on exploring the potential of harnessing the natural power of this immune defense. These most recent reports allude to functions of the A3 proteins that extend beyond their well-characterized designation as restriction factors. The emerging story implicates the A3 family as not only defense proteins, but also as participants in the broader innate immune response.

## 1. Introduction

 In 2002, the cloning of *APOBEC3G* (*A3G*; then called *CEM15*) and the identification of the protein product of this gene as the first cellular protein capable of restricting HIV-1 infection revealed a novel direction for chemotherapeutic intervention and ignited the search for additional defense proteins capable of counteracting viral invasion [1]. The report of this cloning solved a long-standing enigma in the field of HIV-1 pathogenesis. Early work examining and comparing the pathogenesis of wild-type and Vif-deficient HIV-1 had yielded conflicting results with some laboratories concluding that Vif was dispensable for productive infection while other groups maintained that Vif expression was essential [2–4]. Ultimately, it was decisively shown that the requirement for Vif was cell-type dependent; permissive cells supported the growth of HIV-1Δ*vif* while nonpermissive cells limited such viral replication [5, 6]. Most interesting and relevant was the inability of Vif-deficient HIV to productively infect primary CD4+ T cells, one of the critical natural targets of HIV-1 infection [2, 3, 5, 7, 8]. The molecular explanation for the “Vif phenotype” remained unexplained for the subsequent decade. Proffered in this early work was the idea that permissive cells expressed a cellular factor that compensated for Vif. An equally valid suggestion was that nonpermissive cells harbored an inhibitory activity of HIV-1 that was itself overcome by the Vif protein. It was subsequently established, in a pair of elegant experiments utilizing heterokaryons formed from fusion of nonpermissive and permissive cell lines that, in fact, nonpermissive cells expressed an activity that suppressed HIV-1Δ*vif *replication [9, 10]. The genetic relatedness of two T lymphocyte lines, one nonpermissive and the other permissive, was exploited in a classical subtractive hybridization experiment; *A3G* was identified as this described suppressive activity. It was found to be almost exclusively expressed by nonpermissive cells and its stable expression in permissive cells conveyed the ability to resist an HIV-1 challenge [1].

 It was quickly appreciated that *A3G* was but one family member of a previously identified gene locus [11]. Subsequent investigation also revealed that A3G exhibited a potent DNA-mutating ability [12]. In humans, seven family members within the locus have been identified; rhesus macaques, the nonhuman primate that serves as the most important animal model for HIV treatment and vaccine testing, also have seven *APOBEC3* genes, while the murine genome contains a single *A3* gene [13–15]. In each of these organisms, the role the *A3 *genes play in counteracting viral invasion is critical. All seven *A3* family members identified in humans exhibit powerful suppressive activity against a range of viruses while the homologous proteins in mice and primates appear to perform similar functions [16–18]. While A3 inhibitory activity is relatively broad, the most well-characterized and studied function is their striking ability to restrict retroviral infection [19]. In an evolutionary response to this restriction, the retroviruses have countered with a battery of genes exquisitely fine-tuned to overcome these endogenous defense proteins.

## 2. The Laboratory Setting

 With one exception (A3C), each of the seven A3 family members in humans has been observed to be capable of combating HIV-1 [1, 17, 20–27]. Whether the antiviral activity observed is relevant during the course of a natural HIV-1 infection has not been unequivocally established for any of the family members and there are valid concerns raised in the interpretation of various data regarding levels of protein expression and potency. However, it is becoming increasingly clear that understanding the battle that is waged between the innate immune system and HIV-1 during acute infection is imperative and the A3 proteins are critical players in this initial encounter.

 While the relative potencies of individual A3 family members in the setting of a natural infection have been difficult to assess, it has been convincingly established that, in the tissue culture setting, A3G exhibits the most potent activity against HIV-1. In a variety of cell types, both primary cells and established cells lines, and under varying experimental conditions, including both single-round infectivity assays and multiple-round replication assays, A3G suppresses the infectivity of HIV-1. HIV-1 Vif has evolved to counteract this impressive activity of A3G by preventing virion encapsidation of this host factor [28–35]. Vif acts as an adapter protein bridging A3G and a Cullin5-elongin B/C-Rbx ubiquitin ligase [36]. Within this complex A3G is ubiquitinylated and subsequently degraded in the 26S proteasome [36, 37]. Other modalities involving Vif prevention of A3G encapsidation have also been documented [28, 32, 34]. Interestingly, dominance of A3G over Vif has been noted under conditions of elevated and/or stabilized expression [1, 34, 36]. This ability to suppress HIV-1 even in the presence of Vif is noteworthy as it has distinct implications for the development of chemotherapeutics designed to interfere with the A3G : Vif axis.

 The anti-HIV-1 functionality of A3G is multifaceted. Its most extensively characterized anti-HIV-1 function is its ability to catalyze cytidine deamination of HIV-1 DNA on the minus strand resulting in the detection of guanosine-to-adenosine transition mutations in reverse transcripts; upwards of 10% of guanosines may be mutated leading to the labeling of this A3G-mediated process as *hypermutation* [34, 38, 39]. The fate of such hypermutated transcripts is not well understood, but certainly this dramatic mutational burden effectively short-circuits viral infection.

 Work from multiple groups has also uncovered deamination-independent anti-HIV effects of A3G that are seen during viral infection [22, 40–49]. The characterization of this editing-independent antiviral function has suggested a block to viral replication that occurs after entry but before integration. While the molecular details of this deaminase-independent function of A3G remain unclear, defective reverse transcription products are commonly observed, indicating that A3G likely acts during the process of reverse transcription. A more comprehensive understanding of this inhibition will be important. All members of the A3 family contain at least one conserved cytidine deaminase active site (CDA; family members A3B, A3D, A3F, and A3G contain two such domains) composed of the signature sequence His/Cys-X-Glu-X_23–28_-Pro-Cys-X_2_-Cys [11, 15]. Early structure-function analysis of A3G was performed by disrupting these suspected catalytic domains with site-directed mutagenesis [41]. The conserved histidine, glutamic acid, and cysteine resides in both the N-terminal and C-terminal domains of A3G were individually mutated and the resulting proteins were independently examined for their catalytic function as well as their ability to suppress HIV-1Δ*vif* infection. The data clearly indicated that the C-terminal CDA domain was responsible for A3G enzymatic function. Unexpectedly the data also suggested that, under specific experimental conditions, significant anti-HIV-1 inhibition could be imparted in the absence of the characteristic mutagenic activity. Subsequent work in a range of experimental systems has supported these original observations. Controversy over these observations primarily stems from claims that these data have most often been cited in experimental settings using mutant A3G exhibiting elevated expression levels [41, 42, 50, 51]. In attempts to clarify the role of A3G expression levels a number of groups have compared A3G protein expression in transiently transfected cell lines and primary CD4+ T cells/macrophages, reporting that expression levels achieved during transient transfection exceed levels observed in primary cells. However, a few cautionary notes are warranted. A3G that is mutated, for instance, at the critical glutamic acid at residue 259 of the protein, has also been shown to have a more limited ability to block the process of reverse transcription thereby suggesting that distinguishing deamination-dependent and -independent activities may be challenging [16]. Additional support for a pleiotropic antiviral function of A3G is provided by observations in which the A3G phenotype is unaffected in cells that do not express uracil DNA glycosylase 2 or SMUG, enzymes responsible for the removal of uracils from single- or double-stranded DNA [52, 53]. As a significant suppressor of HIV-1, a multipronged ability of A3G to inhibit HIV-1 would have notable benefits to the invaded host.

 Using a variety of cell lines and experimental conditions, the anti-HIV-1 activity of A3B, A3D, A3F, and A3H (haplotypes I, II, V, and VII) has also been conclusively demonstrated [17, 20, 21, 23–25, 27, 54, 55]. Hypermutation is often recorded as coincident with antiviral activity, although, in the case of A3B and A3F, as with A3G, there are observations of HIV-1 suppression in the absence of hypermutation [24, 42, 43]. Sensitivity to Vif regulation has been observed for A3D, A3F, A3G and A3H while A3B and A3H/Haplotype I resist Vif-mediated virion exclusion and thus exhibit detectable activity against wild-type HIV-1 virus. However, not all of these family members are equally likely to contribute to HIV-1 resistance during a natural infection; A3B is primarily expressed in B cells and makes it unlikely that this protein contributes appreciably to inhibition of HIV-1 [17, 20, 21, 23, 24, 56–58]. Similarly, the expression of the A3H/Haplotype I restricts wildtype HIV-1, but the protein is inherently unstable [20, 56]. A question with important clinical implications is whether this intrinsic instability may be overcome while harnessing the natural power to combat wild-type viral infection [58, 59].

Until recently, the role of A3A in HIV-1 inhibition was unappreciated outside of two significant observations: the first being a correlation between its expression in monocytes and the susceptibility of these cells to HIV-1 infection, and the second was that expression of A3A was confined to cells of the myeloid lineage and this expression was positively regulated by INF-*α* [60–62]. Berger et al. have now described a novel and critical role A3A plays in the early phase of HIV infection, specifically in myeloid cells [22]. When primary myeloid cells were infected with HIV-1 and the induction of expression at the A3 locus was examined, it was shown that these cells preferentially induced *A3A*, on both the mRNA and protein levels; induction of other A3 family members in these cells was not detected and A3A induction in peripheral blood lymphocytes was negligible. The induced A3A was protective upon HIV-1 challenge and depletion of A3A in primary macrophages and dendritic cells increased viral replication in both single-round infectivity assays and a spreading infection. Similar to other A3 proteins this viral restriction was primarily observed as a profound suppression in the accumulation of viral DNA suggesting interference with an early step of reverse transcription; limited editing of viral reverse transcripts was detectable, but the evidence suggested that enzymatic function was not the sole antiviral function. Notwithstanding its common role as an *A3* family member involved in HIV-1 control, A3A exerts its antiviral function uniquely. It is not producer cell-derived A3A that impacts virus replication, but rather it is the pool of A3A present in the actual *target* cell itself that inhibits incoming HIV-1 particles. Data from independent laboratories strongly support these conclusions for this role of A3A in target myeloid cells [63–65].

**Figure 1:**
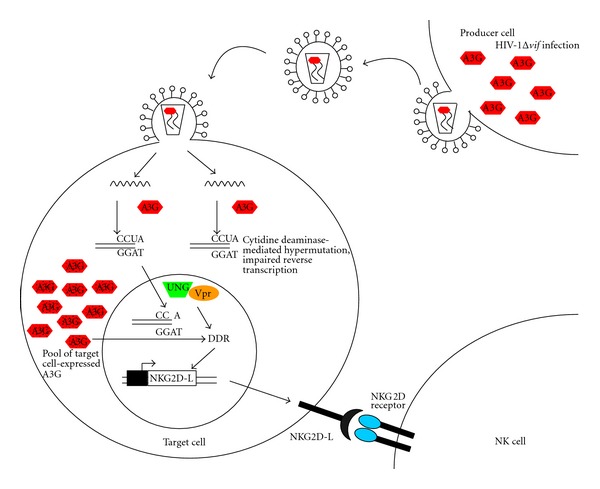
A3G can exert multiple antiviral effects against HIV-1 infection. Virion-packaged A3G restricts HIV-1Δ*vif* replication via cytidine deaminase-mediated hypermutation as well as interfering with efficient reverse transcription. Additionally, the introduction of the uridines into the minus-strand DNA during reverse transcription triggers the DNA damage response (DDR). This induction of DDR involves other proteins, including the host protein, UNG, and the HIV-1 Vpr protein. Among other downstream effects, the DDR stimulates the transcriptional synthesis of NKG2D ligands. The subsequent expression of these proteins on the surface of the HIV-infected cell sensitizes it to NK cell lysis. It should also be noted that A3G expression *within* the target cell (designated as dotted symbols to distinguish it from the virion-packaged A3G). Also critically participates in the DDR activation.

 Within cells of the myeloid lineage, A3A appears to be the critical suppressor, exerting its effect independently of its editing ability. In CD4+ T cells in the tissue culture model of infection, A3G activity dominates, and its inhibitory function is exerted utilizing both editing-dependent and -independent mechanisms. A3A functions in the target cell while A3G functions in the producer cell. Recent observations, however, have now suggested an unexpected and intricate antiviral role played by the A3G expressed in target cells [66]. Expression of either A3A or A3G activate the cellular DNA damage response (DDR) [67]. In the case of A3A, a G_1_/S-phase cell cycle arrest is also induced and its catalytic domain is implicated in the effect. While the relevance of these interesting observations in regard to HIV-1 infection is not immediately obvious (the A3A experiments were performed in human osteosarcoma cells) the role that the DDR response pathway plays in the innate immune response has only recently been explored and appreciated [68, 69]. Experimental observations support the idea that triggering the DDR pathway acts as an alerting mechanism for the innate immune system [66, 68, 70, 71]. In the emerging A3G story this certainly seems to be the case (Figure 1). Norman et al. examined expression of the critical Natural killer (NK) cell-activating ligand, NKG2D-L, in HIV-1-infected primary CD4+ T cells [66]. They compared expression of NKG2D-L under conditions of wildtype HIV-1 infection and HIV-1Δ*vif *infection and found a surprising discrepancy: the combination of Vpr and A3G in the HIV-1Δ*vif* infections activated the DDR ultimately leading to the upregulation of both A3G and *NKG2D-L*. Increased expression of NKG2D-L sensitized the HIV-1-infected cell to NK-mediated killing. In the presence of Vif this NK-mediated killing was blunted. The role of target cell-expressed A3G was further verified using shRNA's targeting A3G mRNA; loss of A3G in an HIV-1Δ*vif *setting resulted in diminished NK-killing and increased (infected) cell survival. The authors suggest that, in a natural infection, the A3G-dependent sensitization of HIV-1-infected cells to NK-mediated killing is hindered by the loss of A3G through Vif-mediated degradation. It bears mentioning that infection of murine primary B cells with the transforming retrovirus Abelson murine leukemia virus (Ab-MuLV) also leads to the induction of activation-induced deaminase (AID) expression [72]. AID is a member of the larger APOBEC-AID family of cytidine deaminases (this grouping includes the founding member, APOBEC1, APOBEC2, APOBEC3A-H and AID). This induction of AID also results in the upregulation of an NKG2D ligand, rendering the infected cells susceptible to NK-mediated lysis. The *in vivo* effect is the profound containment of Ab-MuLV replication and the ability of the host animals to restrict the virus and survive this pathogenic encounter. This indirect effect of AID is also linked to the DDR-stimulated signaling pathways. Details on the mechanistic details of these antiviral functions have not yet been fully characterized. Particularly intriguing is whether the catalytic function of A3G and/or AID is necessary for these effects, and, if so, how is this enzymatic capacity utilized. With the description of the involvement of the DDR, it is suspected that the signature cytidine deaminase modality would be important but confirmation of such speculation is warranted. Based on these observations, therapeutic approaches that interfere with the process of Vif-regulated degradation of A3G could potentially strengthen not only a potent intracellular defense, but also impact the ability of NK cells to attack infected cells.

## 3. The Picture in the Clinic

As astounding as our progress has been in understanding the molecular and mechanistic details of A3 proteins and their interaction with HIV-1, providing data for the *in vivo* relevance of A3 activity has been significantly more challenging. Experiments manipulating A3G in the laboratory have supported the proposition that elevated expression levels of this restriction factor can and do alter wildtype HIV-1 infectivity; clinical correlates of this *in vitro* observation have been more difficult to gather. With few exceptions, the clinical work to date has principally focused on A3G and the effect its fluctuating expression levels and catalytic activity can have on HIV-1 infection and progression. Clinical analyses do not often lend themselves to large sample sets, and the confounding combinatorial effects of host genetics and environment strain efforts of reproducibility. With these openly acknowledged limitations recognized, there remains an increasing amount of suggestive evidence that corroborates the idea that A3G expression and/or activity can modulate natural HIV-1 infection [59, 75, 77, 79–81] (Table 1).

 In infected individuals, hypermutated HIV-1 proviral genomes and elevated A3G expression levels have been correlated with both lower viral loads and increased CD4+ T cells counts [75, 80–83]. In a relatively large study, Land et al. noted the significant association between proviral hypermutation and increased peripheral blood CD4+ T cell count. A3G expression was not directly quantified and the detected hypermutation was used as a surrogate for catalytic function of A3G.

 More direct analysis of *A3G* expression in the setting of a natural HIV-1 infection has also yielded tantalizing hints of A3G control. Working with a small cohort of women, one group recently reported an interesting correlation between individuals expressing higher levels of A3G before HIV-1 infection with the establishment of a lower viral set point after infection [77]. Perhaps the most interesting cohorts in which to examine A3G expression levels and the importance of these levels during viral infection *in vivo* are long-term nonprogressors (LTNPs), elite suppressors (ESs), and highly exposed seronegative (HESN) individuals. To date, there has been no reporting of A3G expression (or activity) as an explanation for the innate ability of an ES to completely control the HIV-1 virus. However, there has been an observation that elevated A3G levels do correlate with higher CD4+ T cell counts and lower viremia within a group of identified LTNPs, suggesting that, under certain conditions, overexpression of A3G may be protective [80]. Two independent studies, examining approximately 67 individuals who have been repeatedly exposed to HIV-1, yet retain their seronegative status, have also presented evidence that elevated A3G expression levels correlate with viral restriction [79, 81]. Amongst these two cohorts a variety of cell types were studied, including PBMCs, CD4+ T cells, CD8+ T cells, CD14+ monocytes, and cervical cells. These cells were assayed for the level of A3G expression primarily determined at the transcriptional level; in a small number of instances, protein expression was also determined. Calculated levels of mRNA and protein in HESN individuals were then compared to both HIV+ individuals and healthy controls and, in both experimental groups, HESN expressed statistically higher levels of A3G expression. One study carried the results further and was also able to show that PBMCs isolated from HESN individuals were able to more effectively limit a wildtype HIV-1 challenge [79]. Interestingly, both PBMCs and CD14+ cells, isolated from these HESN individuals, appeared to exhibit higher responsiveness to IFN-*α* treatment as measured by the induction of A3G expression.

 Finally, a recent experiment utilizing the SIV/macaque model for HIV-1 infection also suggests that investigating and understanding the consequences of increased A3G (and A3F, in this case) expression levels may elucidate the protective role these defense proteins can play *in vivo *[84]. Infected macaques were separated by clinical stage (chronic versus AIDS stage of infection) and compared to uninfected controls. In isolated PBMCs, CD4+ T cells, and peripheral lymph nodes there was a demonstrated negative correlation between *A3F/G* mRNA expression and viral loads. In addition, the difference in A3F/G expression between control and infected animals was even more pronounced when individuals whose disease course mimicked that of HIV-1/LTNPs were specifically compared. One of the novel aspects of this reporting was the kinetic observation of the *in vivo* regulation of *A3G* gene expression after SIV challenge. Seven days after infection *A3G* expression levels began to rise and this expression induction peaked on Day 10 after infection. Peak viremia was measured on Day 14. The concomitant rise of A3G levels, leading the rise of replicating virus levels, suggests that the struggle for control between this intracellular restriction factor and the invading pathogen occurs early, during acute infection. This supports previous reports noting the HIV-1-induction of A3G expression and the critical importance this early encounter may play on establishing viral set point [22, 79, 81, 84–86].

 In spite of the meticulous analyses and important work accomplished, the current clinical understanding of how and whether A3 family members modulate HIV-1 infection is limited and somewhat unsatisfactory. A consensus has not yet emerged and such agreement will likely require a more collaborative and coordinated effort, across cohorts and experimental approaches. The details of designing such experiments are themselves still fraught with unknown parameters; for instance, which cell types and/or tissues should be examined? Is an examination of proviral hypermutation or viral genome editing enough to serve as a marker for A3G antiviral function? Is a measurement of A3G mRNA sufficient to draw conclusions regarding expression of the protein and resultant antiviral activity? At least two groups have noted a disconcerting disconnect between A3G mRNA and protein expression in PBMCs [60, 79]. Do other A3 family members play distinct roles at discrete stages of viral infection? In spite of this minefield of questions and the intrinsic limits placed on a data set as soon as a cohort of study is chosen, ventures into the clinical realm are paramount and it is only this data that can ultimately reveal the role of the A3 family in potentially containing HIV-1 infection.

## 4. The Murine Story

 In contrast to the undetermined impact human A3 proteins have in limiting natural HIV-1 infection, systematic and directed experiments in mice have conclusively shown that murine A3 (mA3) is essential in containing and restricting several murine retroviruses: MMTV, a betaretrovirus (mouse mammary tumor virus), F-MuLV (Friend murine leukemia virus), a gammaretrovirus, as well as FV (Friend virus) [86–88]. Other murine gammaretroviruses, such as MLV (murine leukemia virus), are resistant to mA3 restriction [89–91]. Unlike the complex *APOBEC3* locus in humans, which contains a tandem array of seven genes, the murine genome encodes a single *APOBEC3* gene, *mA3* [11, 92]. The knockout of *mA3* was achieved quickly after the identification and cloning of A3G [93]. While a preliminary examination of the mice was relatively uninteresting, detailed characterization of the response of these animals to specific viral challenge was both illuminating and exciting.

 In a series of informative and elegant *in vivo* experiments, it was shown that MMTV spreads more rapidly and is disseminated more extensively in mice lacking a functional *mA3* gene as compared to wildtype mice. The *mA3* knockout mice exhibited higher initial viral loads and a shorter time to the development of mammary tumors [86]. It was interesting to note that the protection afforded by mA3 was not absolute; mA3 blunted, but did not completely inhibit, MMTV infection, suggesting even partial protection has a significant role in *in vivo* pathogenesis. The molecular mechanism of this mA3-dependent repression of infection remains unidentified, although it does appear that this antiviral function is exerted independently of any detectable hypermutation or viral genome editing. In this setting mA3-mediated containment of MMTV bears a striking resemblance to A3A-dependent control of HIV-1 in myeloid cells: neither inhibition requires a detectable hypermutation function, although the block to viral infection traces to an early post-entry step, and antiviral function is exerted by protein expressed in target cells [22, 86]. In the case of mA3, antiviral function was a combinatorial effect of both virion-packaged and endogenously expressed protein. In terms of potentially harnessing the innate power of the A3 proteins, the most intriguing observation was the reporting that pre-treatment of wildtype mice with either LPS or INF-*α* upregulated mA3 expression in dendritic cells, the first cells infected during MMTV exposure. This early elevation of mA3 expression directly correlated with increased resistance to MMTV. Mice lacking *mA3* were unable to restrict viral infection despite either treatment [94]. This result speaks directly to some of the underlying concerns regarding the detrimental consequences of manipulating the expression of A3G and certainly bolsters the hypothesis that increased expression of this protein could ameliorate restriction of HIV-1 infection.

 Finally, it is interesting to note that in addition to reducing MMTV replication, virion-incorporated mA3 has also been shown to be able to markedly reduce the *transmission* of virus [95]. MMTV, as a number of other retroviruses, including HIV-1, is transmitted vertically through breastfeeding. In an investigation examining the route of transmission, Okeoma et al. report that not only was *mA3* mRNA readily detectable in mammary epithelial tissue but that this packaged mA3 significantly decreased MMTV transmission. In an effort to extend these observations to HIV-1 infection, this group examined expression of the *A3* genes in primary human mammary tissue and found significant levels of both *A3F* and *A3G* mRNA. Whether this expression translates into protection from the vertical transmission of HIV-1 is not yet clear. However, the trajectory of this study is interesting taken in the context of HIV-1 infection in which breastfeeding accounts for approximately 40% of vertical transmission [96].

 FV causes immunosuppression and leukemia in mice. Interestingly, mice strains are differentially susceptible to FV, and a number of genes have been implicated in the resistance to this disease [97]. Both cell-mediated and humoral responses appear necessary for recovery and, naturally enough, the major histocompatibility complex (MHC) locus has been identified as important. However, an essential non-MHC gene, *Recovery from Friend virus gene 3* (*Rfv3*), has also been implicated [98]. Mice strains resistant to FV (e.g., C57BL/6), possess *Rfv3* resistance alleles, develop high concentrations of protective neutralizing antibodies, and recover from viremia. Mice strains susceptible to FV infection (e.g., BALB/c) fail to mount the protective humoral response, develop splenomegaly and erythroleukemia, and succumb to viral infection. In a revealing study, passive immunization of susceptible mice decreased mortality dramatically, suggesting that the *Rfv3* locus critically influences the production of the protective neutralizing antibodies [99].

 The first reporting of the genetic region encompassing *Rfv3* was in 1979 [98]. It was to be 30 years before two groups simultaneously identified *Rfv3* as *mA3* [87, 88]. Using a range of both *in vivo* and *in vitro* experiments they convincingly showed that *mA3* expression was critical to the restriction of FV infection and resulted in the suppression of virus particle infectivity. This inhibition to viral replication occurred after entry, but before integration, presumably affecting an early stage of FV infection (potentially reverse transcription). The description of the restrictive capacity of *mA3* was reminiscent of the extensive data characterizing the A3G anti-HIV-1 function. It should also be noted that, in the FV system, mA3 function was exerted independently of any detectable cytidine deamination activity. While the observations supported the identity of mA3 as the suppressive factor, a consensus on what distinguished a resistant *mA3* allele from a susceptible allele was less discernable. Preliminary data implicated the influence of *mA3* polymorphisms on expression level, essentially suggesting the resistant *mA3* alleles were more highly expressed [87, 88, 100]. In addition, there was also suggestion of an important role for a coinherited B-cell-activating factor receptor (*BAFF-R*) allele [101].

 Recent work probing the resistant versus susceptible *mA3* alleles has supported previous suggestion that an *mA3* splice variant lacking exon 5 may be more potent than a full-length isoform [89, 102]. This latest work suggests that the *mA3*Δexon 5 variant is more efficiently translated and the overall combinatorial effect of elevated mRNA levels and preferential translation of the *mA3*Δexon 5 account for significantly higher levels of mA3 protein capable of potently restricting FV infection [102]. A small number of genetic variants within the *A3* family and their respective relationship to HIV-1 disease acquisition and progression have been described: the H186R variant of A3G is associated with rapid progression in African American populations, the C40693T variant of *A3G*, as well as the homozygous loss of *A3B*, may be associated with increased infection susceptibility, and Haplotype I of A3H may provide resistance to infection [59, 73, 74, 78]. To date, a molecular understanding of how these variants modify (or fail to modify) HIV-1 disease is sorely lacking. Details of the defining characteristics of the resistant *mA3* alleles are of significant interest upon contemplation that such differences, when identified, could be thoroughly dissected in a relevant *in vivo* setting, perhaps providing valuable insight into mechanistic detail. Such details may expand our understanding of the human versions of the *A3* family and the critical polymorphisms.

 What is also underscored in these reports is the importance of characterizing *both* expression levels and allelic differences of individual *A3* genes within this family. Fluctuations of *A3G* mRNA levels, in which *A3G *gene expression is upregulated, have been reported across the immature-to-mature differentiation transition in dendritic cells (DCs) [65, 103]. The ability of mature DC's to resist HIV-1 infection is well documented, and this correlative observation is intriguing [104, 105]. An observation reported in the MMTV system is also suggestive: the DC's of mice stimulated with LPS 24 hours prior to a viral challenge exhibited a modest (3-4-fold) increase in *mA3* mRNA levels, but displayed a significantly increased restriction of MMTV [94]. Finally, a recent paper examining a novel role for A3G in the sensitization of infected cells to NK-mediated lysis suggests that small fluctuations in *A3G* expression levels may have profound functional consequences [66]. These studies are interesting for their suggestion that modest elevations of *mA3* and *A3G* gene expression can lead to impressive increases in viral restriction.

## 5. Concluding Remarks

 The unfolding story of the multifunctional characteristics of the A3 family is fascinating. When the identification and characterization of A3G as a potent restriction factor emerged, the field raised numerous important questions and formulated strategies for capitalizing on this natural innate defense. Over several years, the identity of the entire A3 family of proteins as important innate restriction factors has been established. The ability of A3G to inhibit HIV-1Δ*vif* infection has been analyzed by a significant number of laboratories, but the full complement of molecular details on how it exerts its antiviral function has not yet been gathered. Cytidine deamination undoubtedly occurs in the setting of a natural viral infection, but it is not entirely clear whether this enzymatic function is the only modality through which A3G can obstruct HIV-1 *in vivo*. An improved understanding of the details of how antiviral functions are exerted is needed. In addition, the important, and likely critical contribution of additional A3 family members *in vivo*, remains largely uncharacterized, although recent work using a tissue-culture model would suggest that a collaborative effort amongst family members is essential [17]. Utilization of both the MMTV/mA3 and FV/mA3 murine systems may be particularly useful. They are the only *in vivo* models of A3 restriction that currently exist. Alteration of the murine genome is relatively tractable and there is a single *A3* gene in the rodent genome; potentially, *mA3* genetic variants may be assessed in this setting. Other outstanding questions include the determination of whether any of the A3 proteins require cofactors or post-translational modifications to function effectively. An important co-factor for APOBEC1 has been delineated and while there is a preliminary suggestion that A3F/3G antiviral activity requires a co-factor, no specific proteins have been identified to date [106, 107].

 Manipulation of the Vif : A3G interaction is also a viable point of chemotherapeutic intervention. To date only one compound specifically targeting Vif and thereby liberating functional A3G from Vif regulation has been reported; rapidly evolving technology may soon identify others [108]. A more comprehensive understanding of the interface involved in this viral and cellular protein association could identify new target sequences. For instance, recent identification of the transcription factor CBF-*β* as a member of the ubiquitin-ligase complex recruited by Vif to degrade A3G may prove interesting when considering novel drug targets [109]. Liberating A3G from Vif-mediated control has been shown to impact HIV-1 replication *in vitro *and suggests elevated levels of A3G can have a significant impact on the kinetics of viral replication, but whether expression levels of *A3* genes can be modulated *in vivo* remains to be determined. A better fundamental understanding of gene regulation and the important regulatory elements within this family is also essential. To date only one of the promoters within the *A3* family has been identified and characterized [85].

 A more collaborative and concerted effort in the examination of various cohorts is more likely to reveal whether there exist meaningful associations between *A3* genes and the ability to completely resist or partially restrict HIV-1. In light of the recent data being produced in the murine systems, an examination of rapid progressors and various *A3* genetic variants is warranted. Additionally, data sets analyzing *A3G* genetic variants, while relevant and useful, may have missed important information about other family members; the recent findings involving *A3A* would suggest that this gene would also be important to examine in a number of cohorts.

 Expanded roles for members of the A3 family have also been reported. These reports attribute an importance to A3 proteins that extends beyond the relatively simple arena of restriction factor. A3G's participation in marking cells for NK-mediated lysis would expand the reach of the A3 family into induced innate immunity, a series of cellular interactions important in bridging the innate and adaptive responses. Further describing and characterizing this observation will be important as it has potentially important implications for treatment during acute infection and vaccine design. In ten years the field has exploded, from the recognition of a single potential restriction factor (A3G) to an impressive understanding of a family of proteins that influence, modulate, and enhance the innate immune response. It begs the tantalizing question: what will the next decade bring?

## Figures and Tables

**Table 1:** Clinical studies correlating A3 family members and HIV-1 pathogenesis.

A3 Family member	Correlation reported	Identification of cohort	Reference
A3B	Homozygous deletion of gene associated with higher: rates of HIV infection after exposure, viral set point, and rate of disease progression	4216 HIV+ patients pooled from 5 longitudinal cohorts: ALIVE, MACS, SFCC, HGDS and MHCS [73] (US-based studies)	An et al. [74]

A3F and A3G	Level of detectable proviral hypermutations that exhibited A3F/A3G cytidine deaminase signatures associated with higher CD4+ cell count	215 HIV+ female commercial sex workers plus 25 HIV+ women who were infected perinatally (Nairobi, Kenya)	Land et al. [75]

A3F and A3G	Elevated expression of A3F and A3G in PBMCs associated with establishment of lower viral set point	30 women from a well-established [76] cohort of female commercial sex workers (Dakar, Senegal)	Ulenga et al. [77]

A3G	186R polymorphism in African Americans associated with rapid progression to AIDS	2430 HIV+ patients pooled from 5 longitudinal cohorts: ALIVE, MACS, SFCC, HGDS and MHCS [73] (US-based studies)	An et al. [78]

A3G	Elevated expression of A3G in CD14+ cells associated with resistance to HIV-1 infection after exposure	30 HESN individuals (Florence, Italy)	Biasin et al. [79]

A3G	Elevated expression levels inversely associated with viral load in LTNPs	6 uninfected volunteers; 17 HIV+ progressors; 8 HIV+ LTNPs	Jin et al. [80]

A3G	C40693T polymorphism, located within intronic sequences, associated with increased risk of infection	122 HIV-exposed individuals; 69 sero converted after exposure, 53 retained seronegative status (Montreal, Canada)	Valcke et al. [73]

A3G	HESN individuals expressed elevated levels of A3G when compared to healthy controls; elevated levels of A3G associated with higher CD4+ cell count in HIV+ patients	26 healthy controls, 37 HESN individuals, 45 HIV+ patients (Mexico City, Mexico)	Vázquez-Pérez et al. [81]

A3H	Haplotype I associated with protection from HIV-1 infection	70 serodiscordant couples (Florence, Italy)	Cagliani et al. [59]

HESN: highly exposed seronegative; LTNP: long-term nonprogressors.
